# Liquid-infused silicone catheters reduce fungal burden and inflammation in *Candidozyma auris* bladder infections

**DOI:** 10.1128/msphere.00098-26

**Published:** 2026-04-15

**Authors:** Alyssa Ann La Bella, Hope Akegbe, Caitlin Howell, Felipe H. Santiago-Tirado, Ana Lidia Flores-Mireles

**Affiliations:** 1 Department of Biological Sciences, University of Notre Dame 167056, Notre Dame, Indiana, USA; 2 Department of Chemical and Biomedical Engineering, University of Maine, Orono, Maine, USA; 3 Graduate School of Biomedical Science and Engineering, University of Maine, Orono, Maine, USA; Virginia-Maryland College of Veterinary Medicine, Blacksburg, Virginia, USA

**Keywords:** *Candidozyma auris*, CAUTI, liquid-infused silicone, urinary catheters, uropathogens

## Abstract

*Candidozyma auris* is a high-priority, emerging fungal pathogen frequently isolated from urine in healthcare settings. These isolates are often associated with indwelling urinary catheters, a primary risk factor for catheter-associated urinary tract infections (CAUTIs). Despite its clinical prevalence, the mechanisms of *C. auris* colonization and pathogenesis within the bladder remain poorly understood. In this study, we screened *C. auris* isolates from diverse clades using an *in vitro* biofilm model and *in vivo* murine models of uncomplicated UTI and CAUTI. While *in vitro* biofilm formation varied among isolates, the presence of a catheter *in vivo* significantly enhanced fungal burden in the bladder. Notably, one strain (B11103) caused rapid systemic dissemination and mortality. To address this, we evaluated a liquid-infused silicone (LIS) catheter coating, which has previously shown efficacy against other uropathogens. The LIS coating significantly reduced *C. auris* attachment *in vitro* and, crucially, mitigated fungal burden on both the catheter and bladder tissue *in vivo* across all tested strains. For the hypervirulent B11103 strain, LIS catheters also significantly reduced dissemination to the kidneys and bloodstream. Furthermore, cytokine analysis revealed that *C. auris* CAUTI upregulates IL-6, CSF3, and CXCL1; importantly, this damaging inflammatory response was also dampened by the LIS catheter. These findings demonstrate that catheterization potentiates *C. auris* pathogenicity and identify LIS catheters as a promising, antimicrobial-sparing strategy to prevent colonization, systemic spread, and inflammation during *C. auris* CAUTI.

**IMPORTANCE:**

This research addresses the critical public health challenge posed by the emergence of *Candidozyma auris,* elucidating its pathogenesis in the urinary tract, the second-most common yet understudied reservoir. Here, we find that *C. auris* exhibits plasticity in its ability to form biofilms in urine and cause uncomplicated urinary tract infections (UTIs) and catheter-associated UTIs (CAUTIs). Importantly, we show that our liquid-infused silicone (LIS) catheters effectively disrupt this cycle by reducing fungal burden, preventing systemic spread, and dampening the damaging host inflammatory response. This work establishes the urinary tract as a critical niche for systemic entry and provides a validated strategy for infection prevention. Urinary catheters make *C. auris* dangerous, but this liquid-infused silicone coating is fighting back.

## INTRODUCTION

*Candidozyma auris* (formerly *Candida auris*) is a fungal pathogen of critical concern in healthcare settings due to its strong adherence properties, antifungal resistance, and high mortality rates (1–4). Originally isolated from an ear canal in 2009, *C. auris* is now frequently recovered from the axilla, groin, nares, urine, blood, and skin (4, 5). As an emerging pathogen, our understanding of *C. auris* biology and pathogenesis within these distinct host environments remains limited (3, 6, 7). Beyond human hosts, the strong adherence properties of *C. auris* extend to inanimate objects, turning hospital surfaces and medical devices into reservoirs for outbreaks (5–7).

Urinary catheters are one of the most prevalent medical devices, utilized in up to ~25% of hospitalized patients and ~22% in nursing homes, for indications such as urinary retention, anatomic obstruction (e.g., prostatic hypertrophy), surgery, neurogenic bladder dysfunction, or for monitoring critically ill patients (8–10). However, catheterization is the primary driver of catheter-associated urinary tract infections (CAUTIs) (11). CAUTIs are a leading cause of hospital-acquired infection, with risk approaching 100% for long-term catheter users, and are associated with severe complications, including sepsis and mortality, as well as substantial healthcare costs (11–13). Despite affecting the bladder, CAUTI pathophysiology differs from uncomplicated urinary tract infections (uUTIs) (11, 14). uUTIs occur in relatively healthy individuals without urinary tract structural abnormalities or immunocompromising conditions, are frequently caused by uropathogenic *Escherichia coli*, and disproportionately affect women (11, 14, 15). In contrast, CAUTIs affect patients of both sexes and involve diverse, often multidrug-resistant pathogens, including fungi (14).

*Candida* spp., specifically *C. albicans*, are the second-most prevalent cause of CAUTIs (11, 16). Although *C. albicans* rarely causes uUTIs in the dynamic environment of a noncatheterized bladder, the placement of a urinary catheter significantly facilitates infection (11, 14, 16). *C. albicans* establishes catheter-associated infections through Efg1-regulated biofilms, which are anchored by the binding of adhesin Als1 to host fibrinogen (Fg) on the catheter and bladder surface (16, 17). Although *C. albicans* drives the majority of fungal CAUTIs, the contribution of *C. auris* to this burden is undefined.

Recent global surveillance of *C. auris* isolation sources indicates that approximately 15% of 12,996 isolates were recovered from urine (5). By 2024, this proportion rose to nearly 20%, establishing urine as the second-most common source of isolation (5). While it is unclear what proportion of these cases coincided with catheterization, anecdotal evidence suggests a strong association between urinary catheters and multidrug-resistant *C. auris* isolates, a factor that increases mortality risk (5). Consequently, the capacity of *C. auris* to establish bladder infections in the absence of a catheter remains unknown and requires further investigation.

Given the commonality and severity of CAUTIs, significant effort is currently directed toward developing novel catheter coatings and materials (18–20). We previously demonstrated that liquid-infused silicone (LIS) catheters significantly reduced both bladder and catheter microbial burdens in a murine CAUTI model (18). This efficacy was observed across a broad spectrum of pathogens, including *Enterococcus faecalis, E. coli, Pseudomonas aeruginosa, Klebsiella pneumoniae, Acinetobacter baumannii,* and *C. albicans (*18). Mechanistically, the LIS coating inhibits the deposition of host proteins, such as Fg, thereby diminishing the scaffold available for pathogen-biofilm formation (18). Furthermore, as an antimicrobial-sparing therapeutic, LIS catheters represent a promising strategy for the prevention of CAUTIs and other medical device-associated infections.

Motivated by the frequent isolation of *C. auris* from urine and its threat as a nosocomial pathogen, we investigated its pathogenesis within the urinary tract. We screened a diverse library of *C. auris* isolates from various clades and sources using an *in vitro* CAUTI biofilm model. Subsequently, we evaluated two urine isolates with distinct biofilm phenotypes and one skin isolate using a murine model of uUTI and CAUTI. We observed that while the presence of a urinary catheter significantly enhances fungal burden in the bladder, the use of liquid-infused silicone (LIS) catheters mitigates both bladder and catheter colonization and systemic dissemination. Importantly, while *C. auris* strains induce varying degrees of immune activation, the use of LIS catheters effectively suppresses both the local bladder inflammation and the severe systemic cytokine response typically seen during CAUTIs and with aggressive strains like B11103. Ultimately, these findings demonstrate that LIS catheters prevent infection by inhibiting fungal attachment and dampening the associated inflammatory response.

## RESULTS

### Urine is a common site of *C. auris* isolation

**Fig 1:**
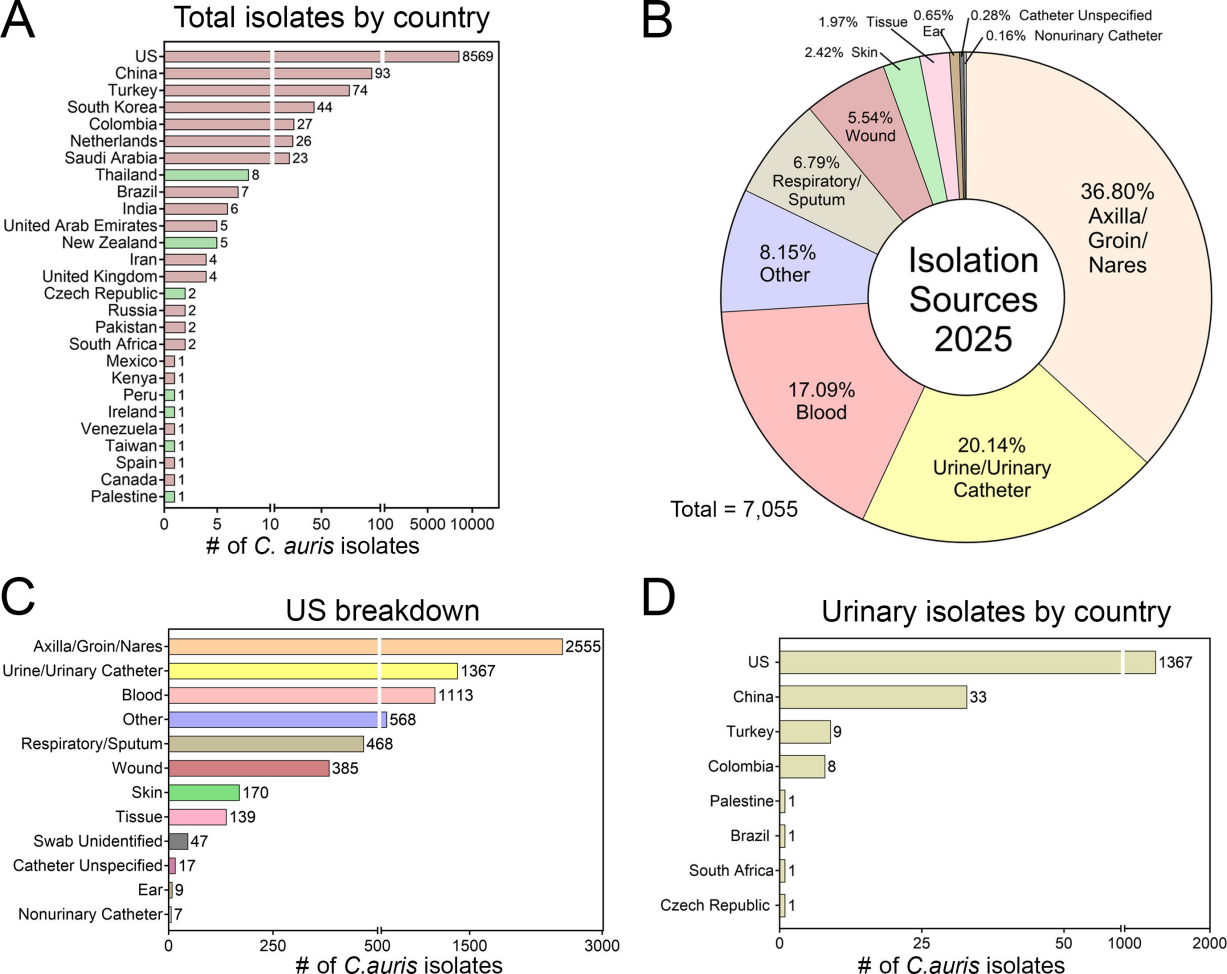
*C. auris* prevalence and isolation sources in 2025. (**A**) The number of *C. auris* isolates in 2025 by country according to the NCBI Pathogen Database. Green bars indicate countries that did not previously have *C. auris* isolates from 2004 to 2024. (**B**) Composition of *C. auris* isolates worldwide in 2025. (**C**) Breakdown of the isolation sources of *C. auris* in the United States. (**D**) Urinary isolates by country in 2025.

Previously, we analyzed metadata from 12,996 clinical strains (2004–2024) and identified urine as a common *C. auris* reservoir, ranking second in the United States and third globally (5). To assess prevalence in 2025, we retrieved 8,912 clinical isolates from NCBI’s Pathogen Database, with the United States reporting the highest volume (Fig. 1A). After excluding 1,810 isolates with unspecified sites and 45 from unidentified swabs, we analyzed the remaining 7,055 strains. Of these, 1,421 (20.4%) were recovered from urine or urinary catheters, maintaining urine as the second-most common source worldwide (Fig. 1B). In the United States specifically, after removing isolates with undefined sources, urine remained the second-most common source among the 6,798 isolates (Fig. 1C). Overall, the United States accounted for the majority of global urinary isolates (Fig. 1D).

### Diverse *C. auris* isolates can form biofilms in CAUTIs *in vitro* conditions

**Fig 2:**
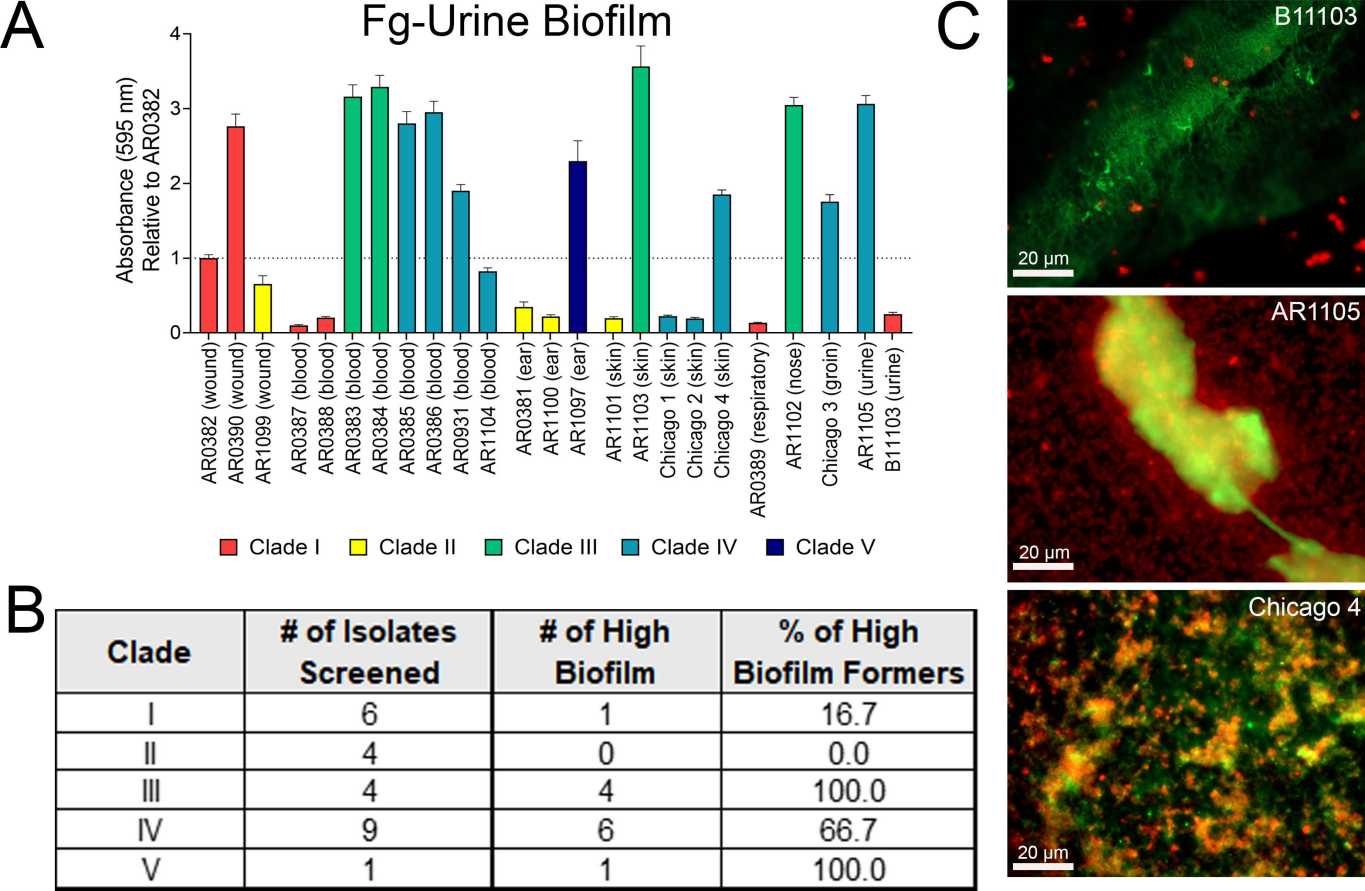
Screening of *C. auris* isolates from different body sites and clades with an *in vitro* catheterized bladder model. (**A**) Biofilm formation of *C. auris* isolates on Fg-coated plastic in urine conditions quantified by crystal violet staining (relative to AR0382). (**B**) Quantification of high biofilm formers (Relative Absorbance > 1) per clade. (**C**) Fluorescent microscopy of fibrinogen-urine biofilms at 40× magnification for three *C. auris* isolates: B11103, Clade I, urine isolate; AR1105, Clade IV, urine isolate; Chicago 4, Clade 4, skin isolate. (*C. auris* red; fibrinogen green). Scale bars represent 20 µm.

Given its prevalence in the urinary tract and on catheters, we evaluated the pathogenic potential of *C. auris* as a CAUTI pathogen. To do this, we assessed biofilm formation using our previously described *in vitro* Fg urine model, which mimics the catheterized bladder environment (16). A library of isolates representing Clades I through V and diverse isolation sources (wound, blood, ear, skin, respiratory, nose, groin, or urine) revealed significant variation in biofilm phenotype (Fig. 2A and B). Biofilm formation was normalized to AR0382 because this strain exhibited an intermediate biofilm phenotype. This baseline provided a mid-range reference point that effectively highlights the variance between robust and poor biofilm-forming isolates. Furthermore, AR0382 has been used by other groups in different model systems, facilitating a comparison across studies (7, 21–23).

In our Fg-urine biofilm model, Clade I and IV displayed greatest heterogeneity, containing both high- and low-biofilm formers (Fig. 2A and B). In contrast, Clade II isolates were consistently low-biofilm formers, whereas Clade III isolates produced robust biofilms (Fig. 2A and B). The single Clade V representative also exhibited a high biofilm-forming phenotype (Fig. 2A and B). Importantly, we observed no correlation between the isolation source and biofilm capacity; both high- and low-biofilm formers were present across all isolation groups (Fig. 2A and B). This was exemplified by two urine isolates, B11103 (Clade I) and AR1105 (Clade IV), which displayed opposing phenotypes. To better understand if biofilm capacity is due to the isolation source or clade, we moved forward with our study characterizing the two urine isolates from different clades (B11103 and AR1105) as well as a skin isolate, Chicago 4, from Clade IV (a gift from Dr. Teresa O’Meara). Fluorescence microscopy confirmed our quantitative biofilm results of these strains, visualizing robust biofilm architecture in AR1105 and the skin isolate Chicago 4, contrasted by a lack of biofilm formation in B11103 (Fig. 2C).

### Urinary catheterization significantly enhances *C. auris* bladder burden *in vivo*

**Fig 3:**
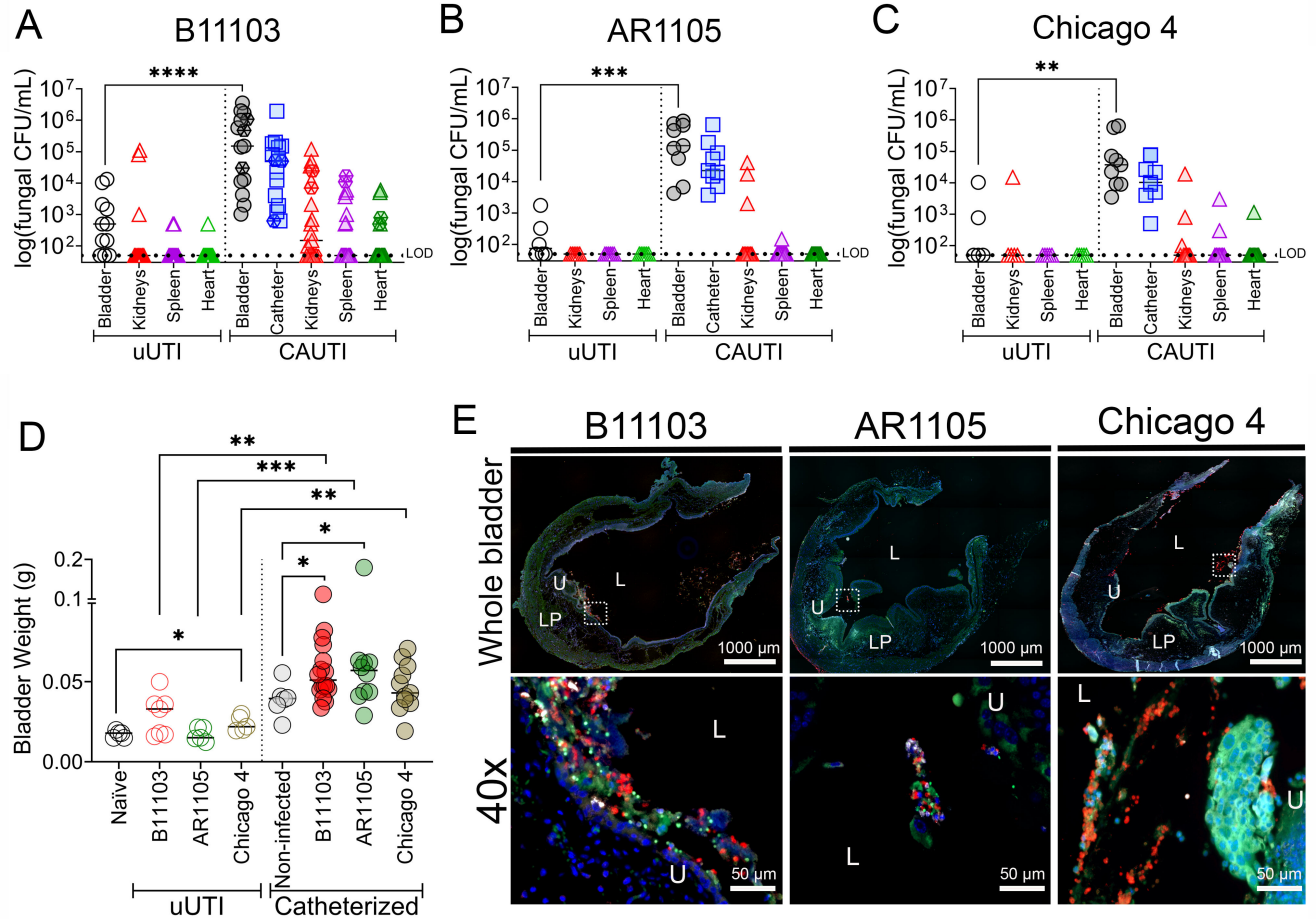
Characterization of *C. auris* pathogenesis in murine models of UTI and CAUTI. Fungal burden of harvested organs in a uUTI and CAUTI mouse model when transurethrally infected with (**A**) B11103, (**B**) AR1105, and (**C**) Chicago 4 after 1 dpi. (**D**) Weights of infected (uUTI) only and catheterized and infected (CAUTI) bladders. (**E**) Immunofluorescence staining of catheterized and infected bladders after 24 h (DAPI blue; *C. auris* in red; fibrinogen in green; Ly6G in white) at 10× (whole bladder) and 40× magnification. Scale bars represent 1,000 µm for whole bladders and 50 µm for 40× images. White box indicates the 40× zoom-in. L, lumen; U, urothelium; LP, lamina propria. Differences between groups were tested for significance using the Mann-Whitney *U* test. *P < 0.05, **P < 0.005, ***P < 0.0005, and ****P < 0.0001.

To determine the uropathogenic potential of *C. auris*, we assessed three isolates (B11103, AR1105, and Chicago 4) at 1-day post-infection (dpi) in established murine models of uUTI and CAUTI (24). For all three strains, the presence of a urinary catheter significantly increased fungal burden in the bladder (Fig. 3A through C). While all isolates successfully colonized the catheter in the CAUTI model, B11103 exhibited exceptional virulence, causing rapid dissemination and mortality, indicated by the hexagon data points with their values in Table S1 (Fig. 3A; Table S1). Notably, we have not observed mortality within 24 h (<1 dpi) with other urinary pathogens, highlighting the exceptional pathogenic potential of *C. auris* in the catheterized bladder. This hypervirulent phenotype extended to the uUTI model, where B11103 caused significantly higher bladder burdens and renal dissemination than either AR1105 or Chicago 4 (Fig. 3A through C). It is also important to note that bladder burden during B11103 CAUTI shows a significant, positive correlation with catheter (*r* = 0.8694), kidney (*r* = 0.8046), and spleen (*r* = 0.5657) fungal burden (Fig. S1A through C). Conversely, AR1105 bladder burden during CAUTIs does not have a correlation with catheter, kidney, spleen, or heart colonization (Fig. S1E through H). Chicago 4 shows a significant, positive correlation with catheter (*r* = 0.7524) burden but lacks a direct correlation with kidney, spleen, or heart colonization (Fig. S1I through L).

As an initial assessment of inflammation in our uUTI and CAUTI models, we used bladder weight as a proxy (Fig. 3D). Compared to naïve (uninfected) controls, uUTI infection increased bladder weights for both strains. This elevation was statistically significant for Chicago 4, while B11103 trended toward significance (Fig. 3D). Urinary catheterization is known to cause physical damage; therefore, to understand the impact of the pathogens, we compared the bladder weights to catheterized non-infected controls. We found the two urinary isolates, B11103 and AR1105, had significantly increased bladder weights compared to the catheterized non-infected bladders (Fig. 3D). Given the high fungal burden associated with catheterization, we visualized colonization via immunofluorescence (Fig. 3E; Fig. S2 for single channels). Imaging revealed that all three strains localized throughout the lumen and lining the urothelium, appearing primarily as single yeast cells or small aggregates that were associated with Fg (Fig. 3E; Fig. S2 for single channels).

### Liquid-infused silicone catheters reduce *C. auris* attachment in urine *in vitro* and mitigate CAUTIs *in vivo*

**Fig 4:**
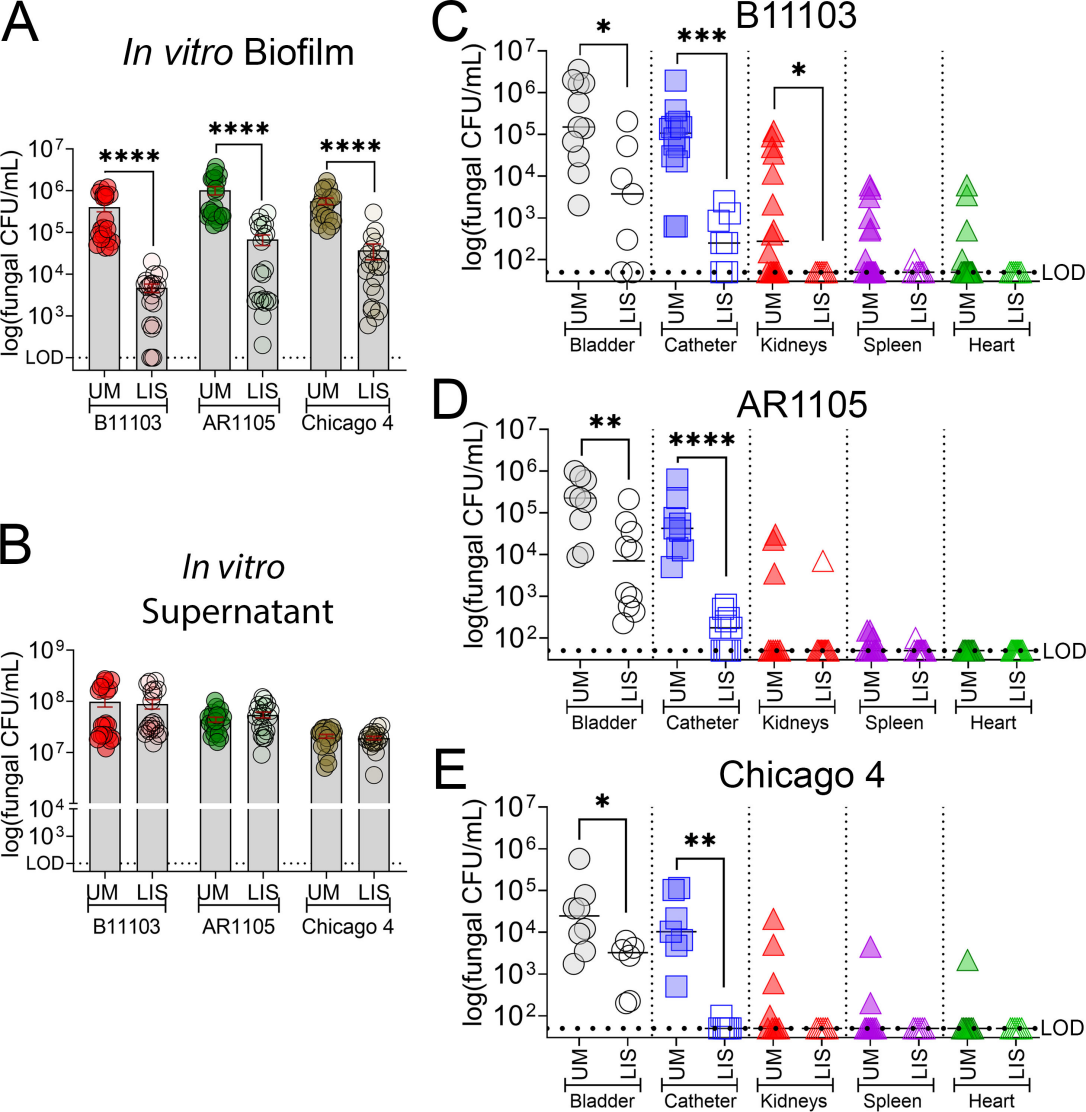
Liquid-infused silicone reduces *C. auris* biofilm formation *in vitro* and mitigates infection severity and urosepsis in a mouse CAUTI model. (**A**) *In vitro* biofilm formation on unmodified (UM) and liquid-infused silicone (LIS) catheter segments incubated in Fg-urine for 48 h. (**B**) Quantification of fungal cells in the urine supernatant of UM and LIS catheters after 48 h. (**C–E**) Comparison of fungal burden in the bladder, catheter, kidneys, spleen, and heart at 24 h post-infection. Mice were implanted with UM or LIS catheters and infected with (**C**) B11103, (**D**) AR1105, or (**E**) Chicago 4. Differences between groups were tested for significance using the Mann-Whitney *U* test. *P < 0.05, **P < 0.005, ***P < 0.0005, and ****P < 0.0001.

Our previous work demonstrated that liquid-infused silicone (LIS) catheters effectively reduce uropathogen attachment, bladder inflammation, and protein fouling, specifically Fg deposition (18). The LIS coating creates a stable, ultrasmooth lubricant layer using silicone oil that eliminates the surface anchors required for microbial adhesins to bind. Given that *C. auris* colonization is driven by a high affinity for abiotic surfaces (6, 7) and colocalization with Fg in the catheterized bladder (Fig. 3E), we hypothesized that LIS catheters would be highly effective against this pathogen. To test this, we quantified adherent fungal cells on unmodified (UM) and LIS catheter segments in our Fg-urine model. After 48 h, the LIS coating significantly reduced biofilm formation across all three *C. auris* isolates (Fig. 4A). Importantly, the planktonic population remained unchanged (Fig. 4B). This dissociation between surface adherence and planktonic growth indicates that LIS catheters function as a passive, anti-adhesive barrier rather than through biocidal activity.

To validate our *in vitro* findings, we assessed the efficacy of LIS catheters *in vivo*. In our mouse CAUTI model, LIS catheters significantly reduced fungal burdens in the bladder and on the catheter surface for all strains (Fig. 4C through E). Notably, catheter colonization was nearly eradicated in the skin isolate, Chicago 4 (Fig. 4E). Furthermore, LIS catheters prevented systemic dissemination, with a statistically significant reduction in kidney colonization observed specifically for the B11103 strain (Fig. 4C). Thus, LIS catheters have been proven to be effective at controlling *C. auris* CAUTI and mitigate the risk of ascending infection and potential urosepsis across diverse clades and isolation sources.

### Differential inflammatory response during *C. auris* uUTI and CAUTI

**Fig 5:**
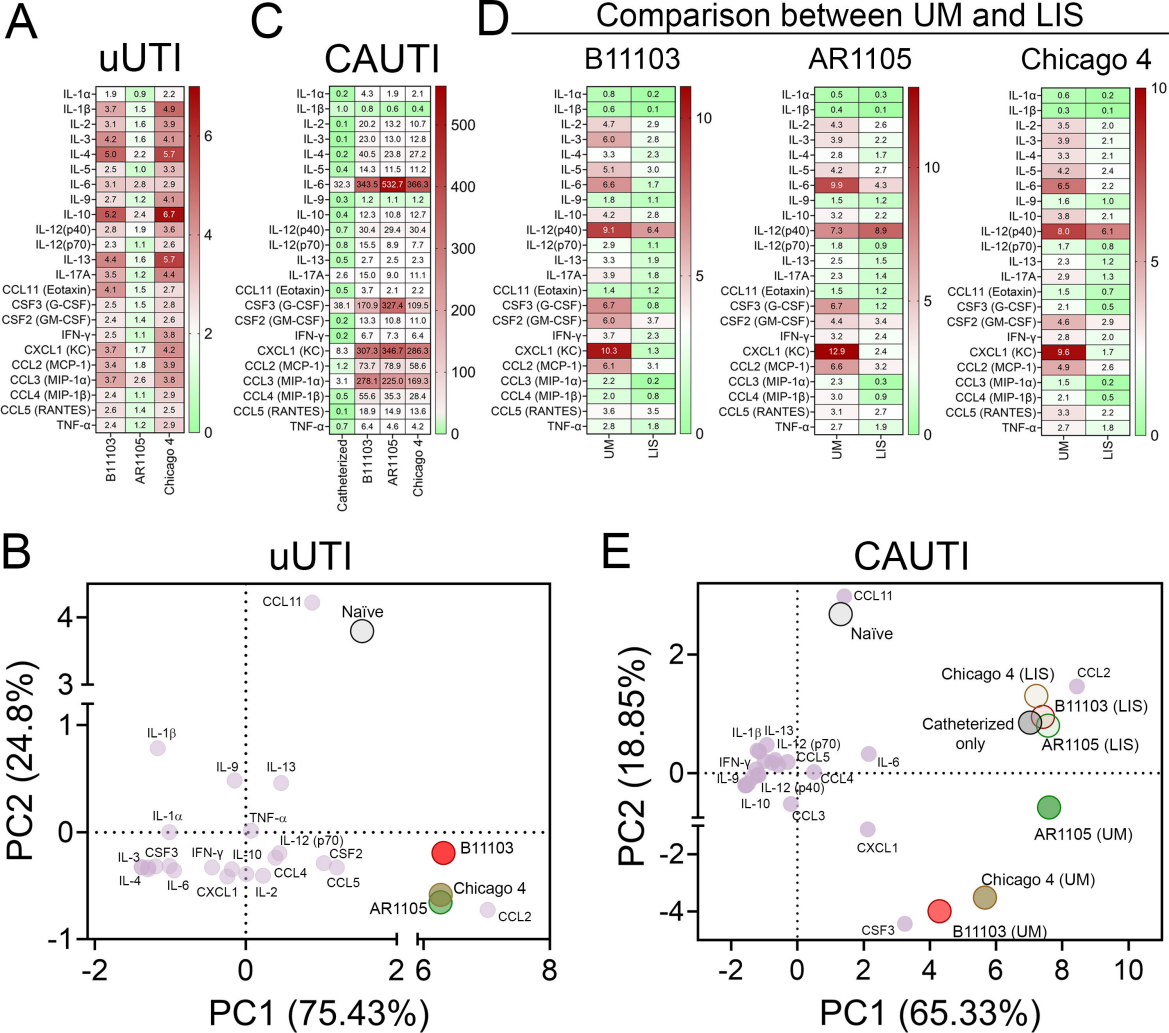
Host-immune response during uUTI and CAUTI. (**A**) Bladder cytokine profiles (fold change relative to naïve controls) for mice with uUTI by strains B11103, AR1105, and Chicago 4. (**B**) Principal component analysis (PCA) biplot comparing the cytokine signatures of these uUTI groups. (**C**) Comparison of cytokine profiles in catheterized-only mice and mice infected and catheterized with UM catheters. Data are normalized to naïve bladder controls to isolate the effect of catheterization and infection. (**D**) Bladder cytokine profiles of mice infected with B11103, AR1105, or Chicago 4 and catheterized with UM or LIS catheters. Data were normalized to the catheterized-only control group to compare the efficacy of UM versus LIS catheters. (**E**) PCA biplot comparing cytokine signatures of naïve and catheterized-only controls against mice infected with strains B11103, AR1105, or Chicago 4 (UM vs LIS).

Current knowledge regarding *C. auris* immunopathology is largely limited to skin and bloodstream infection models (25, 26). To define the local response in the urinary tract, we assessed the levels of 23 cytokines in bladder tissue harvested from uUTI and CAUTI mice at 1 dpi. During uUTI, cytokine levels were calculated by comparing fold change over naïve (non-infected and non-catheterized) bladders, finding that B11103 and Chicago 4 induced a higher cytokine response than AR1105 (Fig. 5A). This correlates with the higher bladder weights (Fig. 3D). Principal component analysis (PCA) revealed two distinct clusters of biological states (non-infected and infected) based on cytokine signatures (Fig. 5B). AR1105 elicited a mild inflammatory response, whereas bladders infected with B11103 and Chicago 4 exhibited significantly higher levels of cytokines, including CCL2 (MCP-1), CXCL1 (KC), IL-4, IL-10, and IL-13 (Fig. 5A and B).

Consistent with previous reports that urinary catheterization alone elicits a strong inflammatory response (13, 18, 27–29), we observed elevated levels of IL-6, CSF3 (G-CSF), and CXCL1 (KC) in catheterized controls compared to naïve bladders. Importantly, during CAUTI, all three strains induced a profile dominated by neutrophil-recruiting signals (including CXCL1, IL-6, CSF3, and CSF2) and monocyte-/macrophage-recruiting signals (including CCL2, CCL3, and CCL4) (Fig. 5C), correlating with the observed bladder inflammation (Fig. 3D). We assessed pathogen-driven responses by calculating cytokine fold changes relative to uninfected catheterized controls. Comparative analysis of UM and LIS groups revealed that LIS catheters significantly reduced inflammatory cytokine levels, even in bladders infected with the highly virulent strain B11103 (Fig. 5D). In contrast to other cytokines, IL-12 (p40) levels remained similar to those in the UM-infected bladders. Consistent with these findings, PCA showed that all UM-infected groups clustered tightly together, characterized by a high-inflammation state driven by pro-inflammatory markers such as CXCL1, IL-6, and CSF3 (Fig. 5E). Importantly, LIS-catheterized infected bladders shift significantly away from the high-inflammation UM-infected bladders’ cluster (Fig. 5D and E). Their cytokine signatures closely resembled those of uninfected catheterized controls (Fig. 5E). These findings correlate directly with the LIS-mediated reduction in fungal burden (Fig. 4D and E), indicating that the LIS coating successfully prevents the infection-induced cytokine storm. In conclusion, LIS technology effectively suppresses inflammation across all *C. auris* tested strains in the CAUTI model.

Given the high systemic dissemination of B11103 observed during catheterization, we analyzed serum cytokine profiles to compare systemic inflammatory responses across strains and between UM- and LIS-catheterized mice. The cytokine data were normalized against serum obtained from catheterized non-infected mice. We found that B11103 elicited the most severe systemic response (Fig. S3A), correlating with the high dissemination rate (Fig. 3A). The cytokine signature was driven by a massive elevation in CSF3, IL-6, IL-1 α, and IL-12(p40) (Fig. S3A). While Chicago 4 and AR1105 also strongly induced CSF3 and IL-12(p40), IL-6 and IL-1α levels were lower than those of B11103 (Fig. S3A through C). Importantly, LIS-catheterized and infected mice showed a consistent reduction in circulating cytokines, specifically reducing IL-6 and CSF3 (Fig. S3A through C). PCA confirmed a differential serum cytokine signature between UM- and LIS-catheterized and infected mice (Fig. S3D). PCA revealed that LIS-catheterized and infected mice shifted toward a distinct immune signature defined by sustained IL-12(p40) and IL-1α (Fig. S3D), rather than a neutrophil-recruiting profile (CSF-3 and IL-6) seen in UM-catheterized and infected mice (Fig. S3A through D). These data demonstrate that LIS technology mitigates strong systemic inflammation driven by CSF3 and IL-6, possibly limiting the local infection burden in the bladder (Fig. 3 and 4).

## DISCUSSION

The emergence of *C. auris* has created a nightmare for healthcare systems. With little knowledge about its basic biology and pathogenesis, researchers have only just begun to elucidate the fungal pathogen’s behavior in various niches. Recent studies have focused on *C. auris*’s skin colonization as well as characterizing bloodstream isolates (3, 23, 26, 30–32). In recent years, urine has become the second-most common isolation source for *C. auris* (Fig. 1) (5); despite the prevalence, there is a critical lack of studies investigating the fungus within the urinary tract environment.

Here, we screened a library of *C. auris* isolates from varying clades and isolation sources in our *in vitro* biofilm model, observing diverse biofilm phenotypes (Fig. 2). Our results identify a possible association between biofilm formation and genetic lineage (clade). However, the considerable intraclade heterogeneity suggests that strain-specific factors may also play a significant role. In our limited sample size, Clade III isolates exhibited robust biofilm potential, contrasting with the low activity of Clade II and the heterogeneity of Clades I and IV. Although confirming these trends requires an analysis of more clinical isolates from all six clades (particularly Clade VI, for which isolates were unavailable for this study), the observed trends provide a preliminary indication of lineage-dependency aligning with the pathogen’s genomic landscape. *C. auris* is characterized by significant genetic diversity, defined by at least six highly divergent lineages. While interclade differences involve thousands of single-nucleotide polymorphisms (SNPs), intraclade diversity arises from smaller variations such as chromosomal rearrangements and aneuploidy, driving diverse resistance and virulence profiles (33). Future studies should focus on dissecting the association between biofilm formation and clade as the inherent interclade isolate variability within our limited sample size precludes definitive conclusions regarding clade-specific phenotypes.

This lineage-dependent pathogenicity mirrors patterns seen in other uropathogens, such as uropathogenic *E. coli* (UPEC). UPEC exhibits significant genetic diversity, with specific phylogenetic groups (e.g., B2 and D) carrying distinct virulence factors, including fimbriae and toxins (34–39). Yet, despite this extensive genetic diversity, UPEC strains exhibit a remarkably conserved gene expression pattern during both human and murine infections (35, 38). Therefore, it is critical to analyze the *in vivo* transcriptional profiles of diverse *C. auris* strains to determine if a similarly conserved transcriptional signature exists during uUTI and CAUTI.

A notable finding in this study is the hypervirulence of the Clade I isolate B11103; however, further studies are necessary to determine if this phenotype is representative of urinary-derived strains or a stochastic characteristic of this specific isolate (Fig. 3; Table S1). While virulence heterogeneity is characteristic of *C. auris*, the rapid mortality observed in our CAUTI model represents a distinct departure from the pathophysiology of other typical uropathogens, including *C. albicans*, where acute lethality in murine models has not been observed (16). Furthermore, we observed a striking inverse relationship between *in vitro* biofilm formation and *in vivo* virulence phenotypes. Specifically, B11103 was a poor biofilm former *in vitro* (Fig. 2), yet it exhibited high colonization in the catheterized bladder, extensive systemic dissemination, and high lethality *in vivo* (Fig. 3A; Table S1). Bladder imaging revealed that *C. auris* yeast cells are able to colonize the urothelium and form biofilms with Fg in the catheterized bladder (Fig. 3E). This discrepancy may be explained by several factors. First, nutritional availability differs significantly; the static *in vitro* model lacks fluid replenishment, potentially limiting fungal proliferation, whereas the catheterized bladder constantly provides fresh urine and serum proteins. Alternatively, biofilm dispersion may play a role. The biofilm might be dynamic, undergoing cycles of attachment and detachment, rather than remaining static. This phenomenon parallels *Proteus mirabilis*, where nutrient limitation induces biofilm dispersion to facilitate spreading (40, 41). Indeed, similar temporal dynamics have been observed in *P. mirabilis* Fg-dependent urine biofilms (42).

Another potential explanation involves an attachment trade-off, a phenomenon well documented in UPEC. In UPEC, the type 1 pilus, specifically the FimH tip adhesin, is critical for successful bladder infection. FimH binds to mannosylated proteins, including uroplakins on urothelial cells and Fg (14). Crucially, successful infection requires FimH to function as a catch-bond (becoming adhesive only under shear stress from urine flow) rather than acting as a static super-glue (43, 44). Analysis of FimH sequences has identified natural mutations that lock the adhesin into a permanent high-affinity state; interestingly, these mutations destroy the catch-bond flexibility (43–45). Consequently, although these mutations confer high mannose binding, they paradoxically lead to reduced UPEC infection capacity (43, 44). This trade-off parallels the behavior of our high-biofilm formers, AR1105 and Chicago 4 (Fig. 2). It suggests that specific mutations may lock adhesins into static conformations; while this increases attachment, it impairs the dynamic detachment-reattachment cycles required for infecting neighboring cells, evading the immune system, and disseminating systemically. This model of impaired dynamic detachment is further supported by our correlation analysis of organ burdens (Fig. S1). While a significant linear relationship between bladder and kidney colonization was observed for the hyper-disseminating strain B11103, this correlation was notably absent in AR1105 and Chicago 4. These data suggest that in high-biofilm-forming strains, dissemination is not a simple linear consequence of primary site burden but is instead limited by an intrinsic, strain-specific attachment-detachment trade-off that restricts systemic spread even when bladder colonization is high.

*C. auris* strains have been famously described as barnacles due to specialized adhesion proteins that facilitate strong attachment to human skin, intravenous catheters, and environmental surfaces (7). However, this raises the question of whether urinary *C. auris* strains are undergoing pathoadaptation, whereby excessive adhesiveness becomes a liability. Instead, a dynamic attachment strategy likely favors immune evasion and systemic dissemination, strategies that are particularly vital in the catheterized bladder since this environment is defined by intense immune surveillance (13, 16, 27, 29). It would be critical to analyze a broad spectrum of urinary clinical strains to identify if this pathoadaptation is happening. This is clinically critical as several studies indicate that patients with *C. auris* candiduria (including those with indwelling urinary catheters) experience worse outcomes, often resulting in mortality (46–48). Therefore, future studies must investigate why certain *C. auris* strains act as localized colonizers, while others cause lethal systemic infection during CAUTI.

Among the three isolates tested, B11103 demonstrated superior bladder colonization in the uUTI model. In the absence of a urinary catheter, most CAUTI-associated pathogens are rapidly cleared from the dynamic bladder environment by the hydrodynamic forces of micturition, limited nutrients, and the immune response (49, 50). Although B11103 colonization did not reach the clinical threshold for uUTI (>10^5^ colony-forming units [CFUs]), it was significantly higher than that of AR1105 and Chicago 4 at 1 dpi. (Fig. 3). This persistence implies that certain *C. auris* strains possess intrinsic uropathogenic traits similar to those of UPEC, independent of catheterization. Future studies using low-dose and temporal models are essential to define the extent of this pathogenicity.

Importantly, the catheterized environment promoted colonization of a skin isolate, Chicago IV (Fig. 3). This, together with the ability of isolates from diverse sources (skin, blood, and urine) to form biofilms in our Fg-urine model, suggests that *C. auris* is a generalist pathogen with high plasticity, capable of adapting to the urinary niche regardless of its isolation origin. This indicates that the catheter acts as a permissive niche, allowing strains to thrive regardless of their isolation origin. This phenomenon parallels findings in *Enterococcus* species, where diverse lineages (including commensal gut strains) are capable of colonizing the catheterized bladder (51).

Remarkably, our LIS catheters significantly inhibited fungal attachment *in vitro* and *in vivo* (Fig. 4). In the murine CAUTI model, these modified catheters drastically reduced both bladder burden and catheter colonization across all three tested strains. Notably, for isolate B11103, the LIS catheter effectively mitigated systemic dissemination, significantly lowering fungal loads in the kidneys, spleen, and bloodstream (Fig. 4). LIS catheters also reduced the exacerbated inflammatory response in the bladder, mitigating a cytokine storm characterized by neutrophil and monocyte-/macrophage-recruiting signals (Fig. 5). Importantly, LIS catheters decreased the damaging response without compromising IL-12 (p40) levels in the bladder or IL-12 (p40) and IL-1α levels in the bloodstream. These cytokines have been shown to provide critical, nonredundant protection against systemic fungal infections by orchestrating innate and adaptive immune responses. IL-12 drives Th1-cell differentiation and IFN-γ production, enhancing macrophage antifungal activity (52, 53), while IL-1α acts as an alarmin to mediate inflammation, reduce tissue damage, and control pathogen growth, particularly in the brain and kidneys (54, 55). Our data suggest that LIS catheters provide a dual benefit: 1) a physical slippery barrier that prevents the initial attachment required for colonization and 2) a modulation of the local immune environment that prevents harmful hyper-inflammation while maintaining protective innate responses. Future studies must now assess the long-term durability of this technology to validate its clinical utility in preventing catheter-associated pathogenesis.

With urine emerging as the second-most common site of *C. auris* isolation, our results establish the catheterized urinary tract as a critical yet overlooked reservoir for pathogenesis. These findings suggest that the urinary catheter acts as a permissive gateway, allowing *C. auris* to establish infection regardless of its isolation source. Crucially, the severe and lethal infection caused by the urinary isolate B11103 highlights the urgent need to determine if urinary strains are undergoing pathoadaptation. Given the highly adhesive nature of *C. auris* and its propensity to colonize medical devices, preventing initial attachment is a critical strategy for reducing infection outbreaks. Our validation of LIS catheters offers a robust countermeasure by inhibiting catheter colonization and subsequent systemic dissemination, while simultaneously mitigating the cytokine storm without compromising protective IL-12 and IL-1α responses. LIS technology addresses both the physical and immunological challenges of CAUTI. Ultimately, this work redefines urinary *C. auris* not merely as a localized colonizer but as a significant threat for systemic disease, positioning surface-modified catheters as an essential preventative strategy in clinical settings.

## MATERIALS AND METHODS

### Isolation source analysis

Clinical *C. auris* isolates uploaded to the NCBI Pathogen Database during the 2025 calendar year were evaluated. Isolates were classified into 12 distinct source categories based on the previously described methodology (5): axilla/groin/nares; urine/urinary catheter (urinary); blood; skin; wound; respiratory tract/sputum; ear; tissue (e.g., bone and various tissue types); unspecified catheter; non-urinary catheter (e.g., central venous lines, dialysis catheters, nephrostomy tubes, and lumbar punctures); others; and unknown/not stated.

### Urine collection

Human urine from healthy female donors was used in this study. Donors had no history of kidney disease, diabetes, or recent antibiotic treatment. Urine samples were pooled from at least two donors, sterilized using a 0.22-μm filter (VWR, 29186-212), and the pH was adjusted to 6.0–6.5. The pooled urine was used immediately for all assays.

### Fungal cultures

Strains utilized in this study (Table S2) were cultured at 37°C with aeration in 5 mL of YPD broth (1% yeast extract [VWR J850-500G], 2% peptone [VWR J636-500G], and 2% dextrose [VWR BDH9230-500G]). For *in vivo* murine models, *C. auris* inocula were prepared by growing the strains statically overnight in 10 mL of YPD.

### Biofilm formation assays

Biofilm formation assays were performed based on established protocols (16). Briefly, 96-well flat-bottom plates (VWR, 10861-562) were coated overnight at 4°C with 100 μL of Fg (150 μg/mL). Fungal inocula, prepared as described above, were normalized to ~1 × 10⁶ CFU/mL and diluted 1:100 in human urine containing 10% heat-inactivated human serum. The diluted suspensions were then added to the coated 96-well plates and incubated at 37°C for 48 h under static conditions.

### Crystal violet staining

Biofilm biomass was quantified using a standard crystal violet assay (16). After supernatant removal, wells were stained with 200 μL of 0.5% crystal violet for 15 min. Plates were washed with water to remove the unbound dye, and the remaining stain was solubilized in 200 μL of 33% acetic acid for 15 min. A 100 μL aliquot from each well was transferred to a new plate, and absorbance at 595 nm was measured using a SpectraMax ABS microplate reader (Molecular Devices). Data were normalized to the AR0382 control strain.

### Fibrinogen biofilm formation and visualization

Biofilm formation and visualization was done as previously described in reference 16. Briefly, a no. 0 cover glass glass-bottom 35-mm petri dish with a 14-mm microwell (MatTek, P35G-0-14-C) was used. Fibrin fiber/nets were formed by adding 100 μL of Fg (1 mg/mL) in PBS and then 10 μL of thrombin (2 U/mL) (Sigma-Aldrich, T6884-250UN) to polymerize Fg into fibrin. Coated dishes were incubated at 37°C for 1 h, followed by overnight incubation at 4°C.

*C. auris* strains were grown as described above, and the inoculum was normalized to ~1 × 10^6^ CFUs/mL in PBS. These cultures were then diluted (1:100) into human urine, added into fibrin-coated dishes, and incubated at 37°C for 48 h under static conditions. After incubation, the biofilms were fixed and incubated with primary antibodies (rabbit anti-*Candida* and goat anti-Fg) for 2 h, followed by a 1-h incubation with secondary antibodies (Alexa Fluor 594-labeled donkey anti-rabbit and Alexa Fluor 488-labeled donkey anti-goat). Biofilms were visualized using fluorescence microscopy, and image analysis was performed using Zen Pro and Fiji (ImageJ) software.

### *In vivo* mouse infection models

Female wild-type (WT) C57BL/6 mice (approximately 6 weeks old) were obtained from The Jackson Laboratory.

#### For CAUTI model

As previously described (24), mice were anesthetized by isoflurane inhalation and implanted with a 6-mm silicone catheter (UM catheters; Braintree Scientific, SIL 025) or a modified liquid-infused silicone (LIS) catheter. Briefly, the oil was absorbed by the silicone tube to create a fully infused silicone tube with a slippery surface (18). Immediately following implantation, mice were transurethrally inoculated with 50 μL of the specified fungal strain (~1 × 10⁶ CFU/mL in PBS). At 1 day post-infection (dpi), mice were euthanized by cervical dislocation under anesthesia. The catheter, bladder, kidneys, spleen, and heart were aseptically harvested. Bladders were weighed and either homogenized for CFU enumeration or fixed overnight in 10% formalin prior to paraffin-embedding, sectioning, and imaging. The remaining organs were homogenized, and catheters were sectioned and sonicated for CFU quantification.

#### For uUTI model

Anesthetized mice were transurethrally inoculated with 50 μL of the fungal suspension (~1 × 10⁶ CFU/mL) without catheterization, as previously described (24). Mice were euthanized at 1 dpi, and organs were processed as described for the CAUTI model.

### Immunohistochemistry of mouse bladders

Mouse bladders were fixed in 10% formalin overnight, before being processed for sectioning and staining, as previously described (56). Briefly, bladder sections were deparaffinized, rehydrated, and rinsed with water. Antigen retrieval was accomplished by boiling the samples in sodium citrate, washing in tap water, and then incubating in 1× PBS three times. Sections were then blocked (1% BSA and 0.3% Triton X-100 [Acros Organics, 21568-2500] in 1× PBS), washed in 1× PBS, and incubated with appropriate primary antibodies (goat anti-Fg, rabbit anti-*Candida*, and rat anti-Ly6G) diluted in blocking buffer overnight at 4°C. Next, sections were washed with 1× PBS, incubated with secondary antibodies for 2 h at room temperature, and washed once more in 1× PBS before Hoechst dye staining. Secondary antibodies for immunohistochemistry were Alexa Fluor 488 donkey anti-goat, Alexa Fluor 550 donkey anti-rabbit, and Alexa Fluor 650 donkey anti-rat. All imaging was done using a Zeiss inverted light microscope. Zen Pro and ImageJ software were used to analyze the images.

### *In vitro* catheter biofilms

Catheter biofilms performed *in vitro* were done as described in reference 18. Disks of UM-silicone (Nalgene 50 silicone tubing, Brand Products) or LIS were cut using an 8-mm leather hole punch. LIS disks were stored in filter-sterilized silicone oil at RT. Disks were skewered onto needles (BD) to hold them in place and put in 5-mL glass tubes (Thermo Scientific) and UV-sterilized for >30 min prior to use. Five hundred microliters of 150 µg/mL Fg was added to each disk in glass tubes, sealed, and incubated overnight at 4°C. *C. auris* strains were grown as described above, and the inoculum was normalized to ~1 × 10^6^ CFUs/mL in PBS. These cultures were then diluted (1:100) into human urine, added to glass tubes with catheters, and incubated at 37°C for 48 h under static conditions.

After 48 h, a sample of the supernatant (urine) was taken to quantify fungal burden in the supernatant by CFUs. The remaining urine was removed, and the catheter piece was washed three times with 1× PBS. Washed catheter pieces were placed in 1 mL of 1× PBS and sonicated for 30 min. A sample from the PBS was taken to quantify biofilm CFUs from the catheter.

### Cytokine analysis

Cytokine quantification and analysis was done as described in reference 13. Bladder samples from mice infected (UTI), catheterized, and infected with *C. auris* B11103, AR1105, or Chicago4 (CAUTI) or catheterized and mock-infected with PBS for 24 h were frozen at −80°C until time of assay. Blood serum samples from mice catheterized and infected with *C. auris* B11103, AR1105, or Chicago4 (CAUTI) or catheterized and mock-infected with PBS for 24 h were frozen at −80°C until time of assay. Bladder homogenates were thawed on ice, microcentrifuged at 11,000 × *g* for 10 min, and supernatants were transferred to a new tube. Samples were analyzed using a Bio-Plex Multi-Plex Assay Kit from Bio-Rad Laboratories (Bio-Plex Pro Mouse Cytokine 23-plex Assay #M60009RDPD) following the manufacturer’s protocols. Fold change over naïve bladder or blood serum was calculated for each cytokine.

### Statistical analysis and reproducibility

Data from at least three experiments were pooled for each assay. Two-tailed Mann-Whitney U tests were performed with GraphPad Prism 5 software (GraphPad Software, San Diego, CA) for all comparisons of biofilms, bladder weights, and mouse models. Values represent means ± SEM derived from at least three independent experiments (*P < 0.05; **P < 0.005; ***P < 0.0005; ****P < 0.0001; and ns, difference not significant). For correlation analysis, log_10_-transformed CFU values for bladders, catheters, kidneys, spleens, and hearts were plotted, and *r* (Pearson’s correlation coefficient), *R*^2^, and *P* values were calculated. For cytokine heatmaps, values represent the median fold change over naïve samples from at least four independent mouse samples. Principal component analyses were performed, and the PC scores of PC1 and PC2 were plotted for both cytokine (variable) and strain and infection type (loadings).

## SUPPLEMENTAL MATERIAL

Supplemental Material msphere.00098-26-s0001.docx Supplemental figures and tables.

ASM does not own the copyrights to Supplemental Material that may be linked to, or accessed through, an article. The authors have granted ASM a non-exclusive, world-wide license to publish the Supplemental Material files. Please contact the corresponding author directly for reuse.
