# Supplementary material for: Liquid-infused silicone catheters reduce fungal burden and inflammation in Candidozyma auris bladder infections
Source: mSphere. 2026 Apr 15;11(5):e00098-26. doi: 10.1128/msphere.00098-26 (PMC13203984; doi:10.1128/msphere.00098-26)
Supplement: Supplemental Material — Supplemental figures and tables. [file msphere.00098-26-s0001.docx]

Liquid-Infused Silicone Catheters Reduce Fungal Burden and Inflammation in *Candidozyma auris* Bladder Infections.

Figures and Supplementary Information

Liquid-Infused Silicone Catheters Reduce Fungal Burden and Inflammation in *Candidozyma auris* Bladder Infections.

Alyssa Ann La Bella^1^, Hope Akegbe^1^, Caitlin Howell^2,3^, Felipe H. Santiago-Tirado^1^, Ana Lidia Flores-Mireles^1*^

^1^ Department of Biological Sciences, University of Notre Dame, Notre Dame, IN, 46556, USA

^2^ Department of Chemical and Biomedical Engineering, University of Maine, Orono, ME, 04669, USA

^3^ Graduate School of Biomedical Science and Engineering, University of Maine, Orono, ME, 04669, USA

*Corresponding author: [afloresm@nd.edu](mailto:afloresm@nd.edu)

Key words: *Candidozyma auris,* CAUTI, liquid infused silicone, urinary catheters, uropathogens


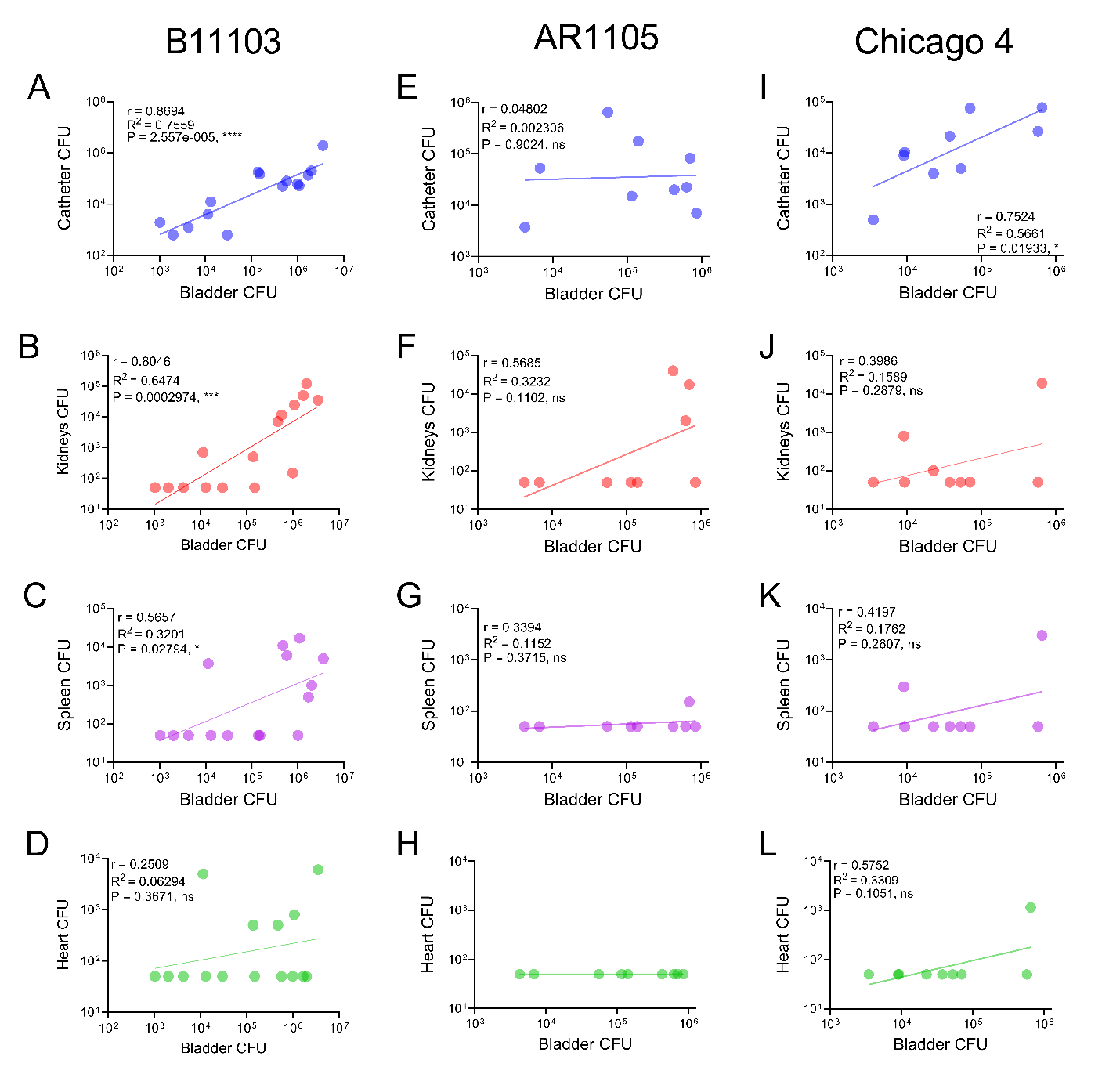


**Supplemental Figure 1. Correlation of bladder burden with catheter colonization and dissemination during *C. auris* CAUTI.** Pearson’s correlation statistical analysis was used to quantify the association between bladder burden and catheter, kidneys, spleen, or heart colonization during B11103 **(A-D)**, AR1105 **(E-H)**, and Chicago 4 **(I-L)** CAUTIs. r, Pearson’s correlation coefficient; R^2^, Coefficient of Determination. *, P < 0.05 was considered statistically significant. **, P < 0.005; ***, P < 0.0005, ****, P < 0.0001.


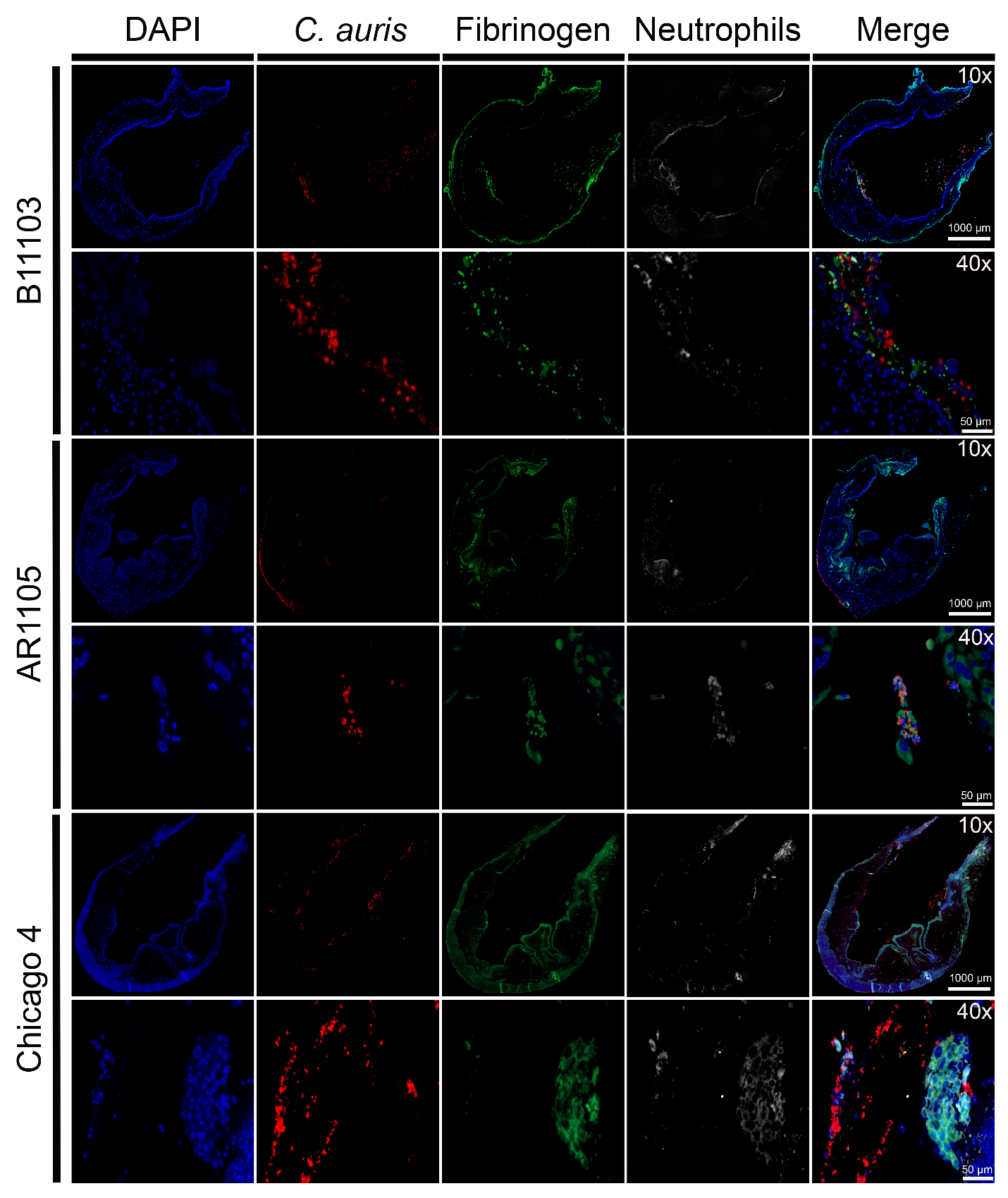


**Supplemental Figure 2.** ***Candidozyma auris* bladder colonization during CAUTI.** Mice were implanted and infected with 1x 10^6^ CFUs with strain B11103, AR1105, or Chicago 4. At 24 hpi, bladder tissues were harvested, fixed, and parafilm-embedded. Bladders were subjected to IF analysis using antibodies to detect Fg (anti-Fg; green), *C. auris* (anti-Candida; red), and neutrophils (anti-Ly6G; white). Staining with DAPI (blue) delineated the urothelium and cell nuclei (representative images). White squares represent zoomed-in areas of higher magnification (40x). Scale bars represent 1000 µm for 10x (whole bladder) images and 50 µm for 40x images.


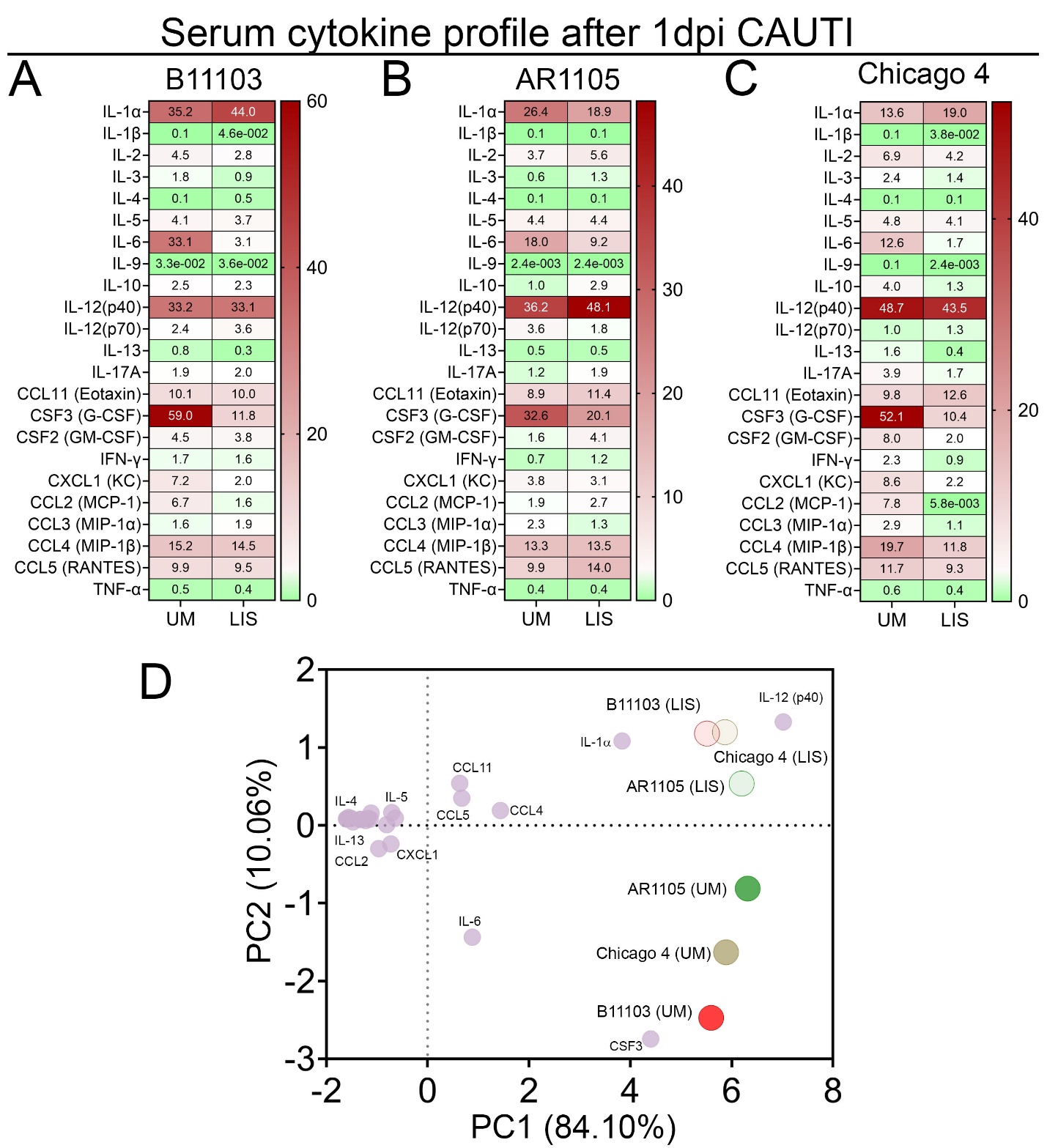


**Supplemental Figure 3.** **Blood serum cytokine profiles during *C. auris* CAUTI with unmodified (UM) or liquid-infused silicone (LIS) catheter.** Blood serum cytokine fold change over catheterized-only bladder levels for mice infected with **(A)** B11103, **(B)** AR1105, and **(C)** Chicago 4. **(D)** Biplot of PCA for UM and LIS catheter samples (large circles) with cytokine loadings (small purple circles) for all three strains.

**SUPPLEMENTAL INFORMATION**

**Table S1. B11103 CAUTI Fatalities Fungal Burden.**

**Table S2. Strains used in this study.**

| **Species** | **Strain** | **Description** | **Reference** |
| --- | --- | --- | --- |
| *Candidozyma auris* | AR0381 | Ear, Clade II | CDC ARisolate Bank ^1^ |
| *Candidozyma auris* | AR0382 | Wound, Clade I | CDC ARisolate Bank ^1^ |
| *Candidozyma auris* | AR0383 | Blood, Clade III | CDC ARisolate Bank ^1^ |
| *Candidozyma auris* | AR0384 | Blood, Clade III | CDC ARisolate Bank ^1^ |
| *Candidozyma auris* | AR0385 | Blood, Clade IV | CDC ARisolate Bank ^1^ |
| *Candidozyma auris* | AR0386 | Blood, Clade IV | CDC ARisolate Bank ^1^ |
| *Candidozyma auris* | AR0387 | Blood, Clade I | CDC ARisolate Bank ^1^ |
| *Candidozyma auris* | AR0388 | Blood, Clade I | CDC ARisolate Bank ^1^ |
| *Candidozyma auris* | AR0389 | Fluid bronchoalveolar lavage/respiratory, Clade I | CDC ARisolate Bank ^1^ |
| *Candidozyma auris* | AR0390 | Pus/wound, Clade I | CDC ARisolate Bank ^1^ |
| *Candidozyma auris* | AR0931 | Blood, Clade IV | CDC ARisolate Bank ^1^ |
| *Candidozyma auris* | AR1097 | Ear, Clade V | CDC ARisolate Bank ^1^ |
| *Candidozyma auris* | AR1099 | Wound, Clade II | CDC ARisolate Bank ^1^ |
| *Candidozyma auris* | AR1100 | Ear, Clade II | CDC ARisolate Bank ^1^ |
| *Candidozyma auris* | AR1101 | Ear, Clade II | CDC ARisolate Bank ^1^ |
| *Candidozyma auris* | AR1102 | Nose, Clade III | CDC ARisolate Bank ^1^ |
| *Candidozyma auris* | AR1103 | Skin, Clade III | CDC ARisolate Bank ^1^ |
| *Candidozyma auris* | AR1104 | Blood, Clade IV | CDC ARisolate Bank ^1^ |
| *Candidozyma auris* | AR1105 | Urine, Clade IV | CDC ARisolate Bank ^1^ |
| *Candidozyma auris* | B11103 | Urine, Clade I | ATCC ^2^ |
| *Candidozyma auris* | Chicago 1 | Skin, Clade IV | Gift from Teresa O’Meara Lab ^3^ |
| *Candidozyma auris* | Chicago 2 | Skin, Clade IV | Gift from Teresa O’Meara Lab ^3^ |
| *Candidozyma auris* | Chicago 3 | Skin, Clade IV | Gift from Teresa O’Meara Lab ^3^ |
| *Candidozyma auris* | Chicago 4 | Skin, Clade IV | Gift from Teresa O’Meara Lab ^3^ |

**REFERENCES**

1 Lutgring, J. D. *et al.* FDA-CDC Antimicrobial Resistance Isolate Bank: a Publicly Available Resource To Support Research, Development, and Regulatory Requirements. *J Clin Microbiol* **56**, doi:10.1128/jcm.01415-17 (2018).

2 *Candida auris Satoh et Makimura (ATCC MYA-5000)*.

3 Pacilli, M. *et al.* Regional Emergence of Candida auris in Chicago and Lessons Learned From Intensive Follow-up at 1 Ventilator-Capable Skilled Nursing Facility. *Clin Infect Dis* **71**, e718-e725, doi:10.1093/cid/ciaa435 (2020).
